# Spatial structure of the microbiome in the gut of *Pomacea canaliculata*

**DOI:** 10.1186/s12866-019-1661-x

**Published:** 2019-12-05

**Authors:** Lan-Hua Li, Shan Lv, Yan Lu, Ding-Qi Bi, Yun-Hai Guo, Jia-Tong Wu, Zhi-Yuan Yue, Guang-Yao Mao, Zhong-Xin Guo, Yi Zhang, Yun-Feng Tang

**Affiliations:** 1 Health Shandong Collaborative Innovation Center for Major Social Risk Prediction and Management, School of Public Health and Management, Weifang Medical University, Weifang, 261053 People’s Republic of China; 2 National Institute of Parasitic Diseases, Chinese Center for Disease Control and Prevention, Key Laboratory of Parasite and Vector Biology, Ministry of Health, WHO Collaborating Center for Malaria, Schistosomiasis and Filariasis, Shanghai, 200025 People’s Republic of China; 3 Community Health Center of Beijing Normal University, Shanghai, 100875 People’s Republic of China

**Keywords:** *Pomacea canaliculata*, Freshwater snail, Gut microbiome, *16S* rRNA gene, High-throughput sequencing

## Abstract

**Background:**

Gut microbes can contribute to their hosts in food digestion, nutrient absorption, and inhibiting the growth of pathogens. However, only limited studies have focused on the gut microbiota of freshwater snails. *Pomacea canaliculata* is considered one of the worst invasive alien species in the world. Elucidating the diversity and composition of the microbiota in the gut of *P. canaliculata* snails may be helpful for better understanding the widespread invasion of this snail species. In this study, the buccal masses, stomachs, and intestines were isolated from seven *P. canaliculata* snails. The diversity and composition of the microbiota in the three gut sections were then investigated based on high-throughput Illumina sequencing targeting the V3-V4 regions of the *16S* rRNA gene.

**Results:**

The diversity of the microbiota was highest in the intestine but lowest in the buccal mass. A total of 29 phyla and 111 genera of bacteria were identified in all of the samples. In general, *Ochrobactrum*, a genus of putative cellulose-degrading bacteria, was the most abundant (overall relative abundance: 13.6%), followed by *Sediminibacterium* (9.7%), *Desulfovibrio* (7.8%), an unclassified genus in the family Aeromonadaceae (5.4%), and *Cloacibacterium* (5.4%). The composition of the microbiota was diverse among the different gut sections. *Ochrobactrum* (relative abundance: 23.15% ± 7.92%) and *Sediminibacterium* (16.95 ± 5.70%) were most abundant in the stomach, an unclassified genus in the family Porphyromonadaceae (14.28 ± 7.29%) and *Leptotrichia* (8.70 ± 4.46%) were highest in the buccal mass, and two genera in the families Aeromonadaceae (7.55 ± 4.53%) and Mollicutes (13.47 ± 13.03%) were highest in the intestine.

**Conclusions:**

The diversity and composition of the microbiome vary among different gut sections of *P. canaliculata* snails. Putative cellulose-degrading bacteria are enriched in the gut of *P. canaliculata*.

## Background

*Pomacea canaliculata*, also known as the golden apple snail, is a large species of freshwater snail originating from South America. Because of its high adaptability, strong fecundity, diverse diet and lack of efficient predators, *P. canaliculata* is widely distributed in tropical and subtropical areas worldwide [1]. Therefore, this species is now considered one of the most invasive alien species in the world and causes serious damage to agriculture and the ecological environment [2]. It is also an intermediate host of *Angiostrongylus cantonensis*, the etiological agent of angiostrongyliasis [3].

Animals sometimes coevolve with the bacteria residing in their gut. Many studies have successfully correlated gut microbiota to host physiology. For example, gut microbiota may play roles in food digestion, absorption and metabolism in humans and other animals [4, 5]; microbiota-derived lactate can activate the production of reactive oxygen species and shorten the lifespan of *Drosophila* [6]; and microbiota can regulate midgut homeostasis to prevent the systemic infection of mosquitoes by inducing the peritrophic matrix [7]. Although the composition and function of gut microbiota have been well studied in humans, several mammals and insects, only limited studies have focused on the gut microbiota of freshwater snails. So far, the microbial community in the guts of *P. canaliculata* snails has not been systematically characterized. Understanding the gut microbiota of *P. canaliculata* might provide insight into the behavior of the host and might be helpful for better understanding the widespread invasion of this snail species. In this study, we investigated the diversity and composition of the microbiota in different gut sections of *P. canaliculata* snails using high-throughput Illumina sequencing targeting the V3-V4 regions of the *16S* rRNA gene.

## Results

### Bacterial complexity of the microbiome in the three gut sections of *P. canaliculata* snails

All 21 snail DNA samples were amplified successfully and sequenced. However, the extraction products of the blank control failed to be amplified by PCR under the same conditions as the snail samples. A total of 1,075,200 valid sequences were acquired from the 21 snail samples, yielding 23,151 valid OTUs at 97% identity. After removing the OTUs with relative abundance less than 0.001%, 2234 OTUs remained and were included in further analysis. The rarefaction curve of observed species reached asymptote (Additional file 1: **Fig. S1**), which indicated that the sequencing depth was sufficient to represent the majority of species richness in each sample.

When analyzed by group, the number of OTUs was higher in the intestine samples (1049.4 ± 184.3) than in the buccal mass (719.0 ± 81.7) and stomach (808.6 ± 189.3) samples (Table 1). Among the 2234 OTUs, 786 (35.5%) were shared by all three groups; 163 (7.0%), 215 (9.6%), and 314 (14.1%) were unique in the buccal mass, stomach, and intestine samples, respectively (Additional file 2: **Fig. S2**).

**Table 1:** Number of OTUs and alpha diversity of the gut microbiome from three gut sections of *P. canaliculata*

Tissues	OTUs	Chao1	ACE	Simpson	Shannon
Buccal mass	719.0 ± 81.7 ^a^	538.5 ± 48.8 ^a^	541.2 ± 54.0 ^a^	0.8641 ± 0.0886	4.517 ± 0.754 ^a^
Stomach	808.6 ± 189.3 ^a, b^	702.6 ± 164.1 ^b^	698.8 ± 158.5 ^b^	0.8911 ± 0.0564	5.231 ± 0.772 ^a,b^
Intestine	1049.4 ± 184.3 ^b^	802.3 ± 153.0 ^b^	804.7 ± 159.0 ^b^	0.9282 ± 0.0402	5.860 ± 0.645 ^b^
F	8.02	7.07	6.93	1.72	6.00
P	0.003	0.005	0.006	0.210	0.003

OTUs, operational taxonomic units; ^a, b^, groups with the same letters indicate no significant difference

The alpha diversity of the gut microbiome was different among the three tissues of *P. canaliculata*. In general, the bacterial diversity was highest in the intestine samples but lowest in the buccal mass samples as assessed by Chao1, ACE, and Shannon indices (Table 1).

### Taxonomic composition of the microbiome in the three gut sections of *P. canaliculata* snails

Among the 2234 OTUs, 99.8% were assigned to the family level, and 48.7% were assigned to the genus level. Finally, 29 phyla and 111 genera were identified from all 21 samples.

**Fig. 1:**
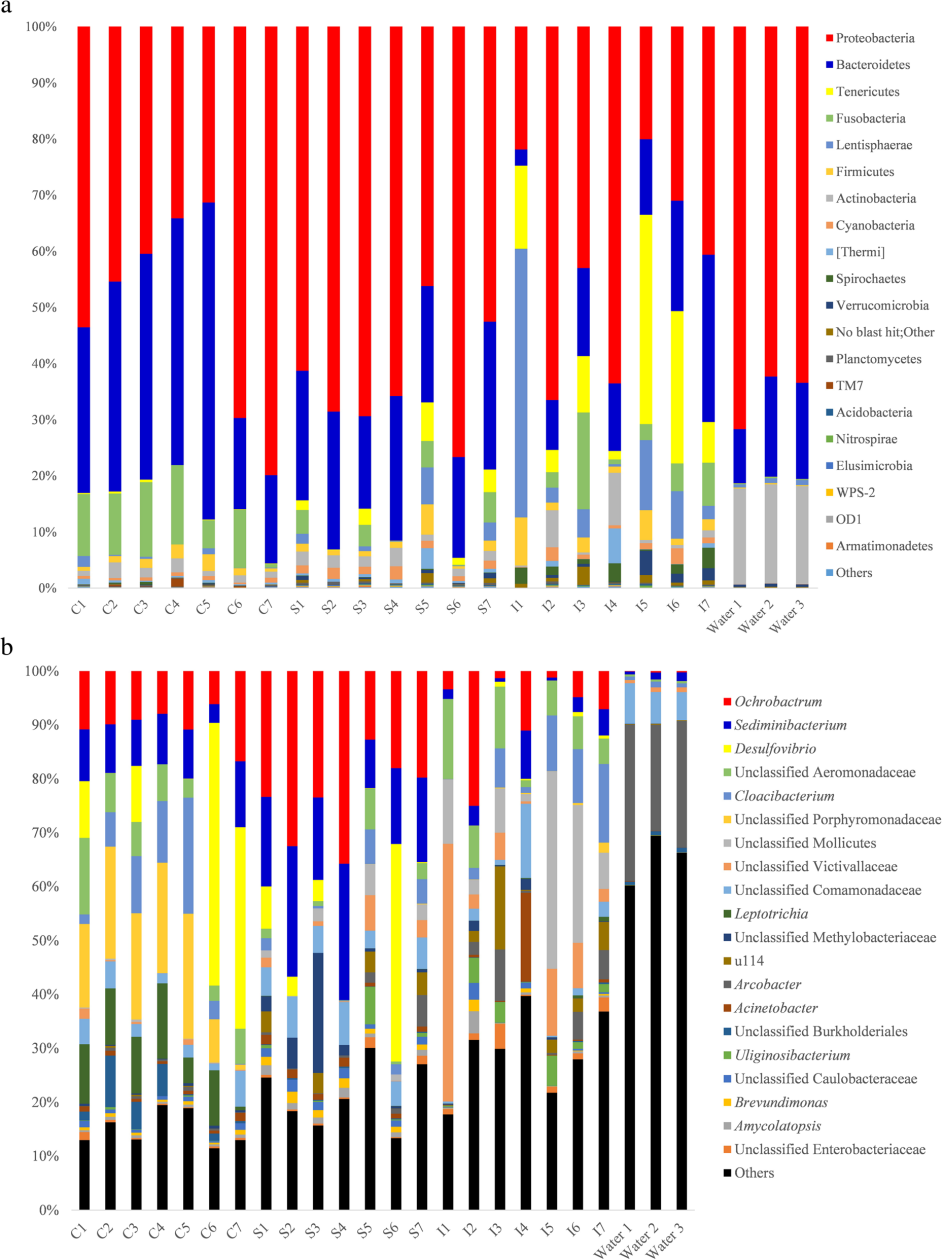
Composition of the bacterial community in the gut of *Pomacea canaliculata* snails (a) at the phylum level; (b) at the genus level. C1 – C7: buccal mass samples; S1 – S7: stomach samples; I1 – I7: intestine samples

At the phylum level, Proteobacteria and Bacteroidetes were the two most dominant phyla in the gut of *P. canaliculata* snails (Fig. 1a), with overall relative abundances of 51.6 and 23.6%, respectively. At the genus level, *Ochrobactrum* was the most dominant genus (overall relative abundance: 13.6%), followed by *Sediminibacterium* (9.7%), *Desulfovibrio* (7.8%), an unclassified genus in the family Aeromonadaceae (5.4%), and *Cloacibacterium* (5.4%, Fig. 1b).

The taxonomic compositions of the microbiome among samples from the different gut sections were diverse. At the phylum level, the relative abundance of Bacteroidetes (mean ± standard error: 34.10% ± 14.72%) and Fusobacteria (9.23% ± 4.76%) was higher in the buccal mass, the abundance of Cyanobacteria (1.55% ± 0.50%) was higher in the stomach, and the abundances of Tenericutes (14.67% ± 13.14%) and Spirochaetes (2.02% ± 1.28%) were higher in the intestine (Additional file 3: **Table S1**). At the genus level, the relative abundances of an unclassified genus in the family Porphyromonadaceae (14.28 ± 7.29%) and *Leptotrichia* (8.70 ± 4.46%) were highest in the buccal mass, *Ochrobactrum* (23.15 ± 7.92%) and *Sediminibacterium* (16.95 ± 5.70%) were highest in the stomach, and two unclassified genera in the families Aeromonadaceae (7.55 ± 4.53%) and Mollicutes (13.47 ± 13.03%) were highest in the intestine (Additional file 4: **Table S2**). Interestingly, the structure of the microbiome in the gut of *P. canaliculata* snails was quite different from that in the water samples (Fig. 1b).

### Similarity of the bacterial community in the three gut sections of *P. canaliculata* snails

**Fig. 2:**
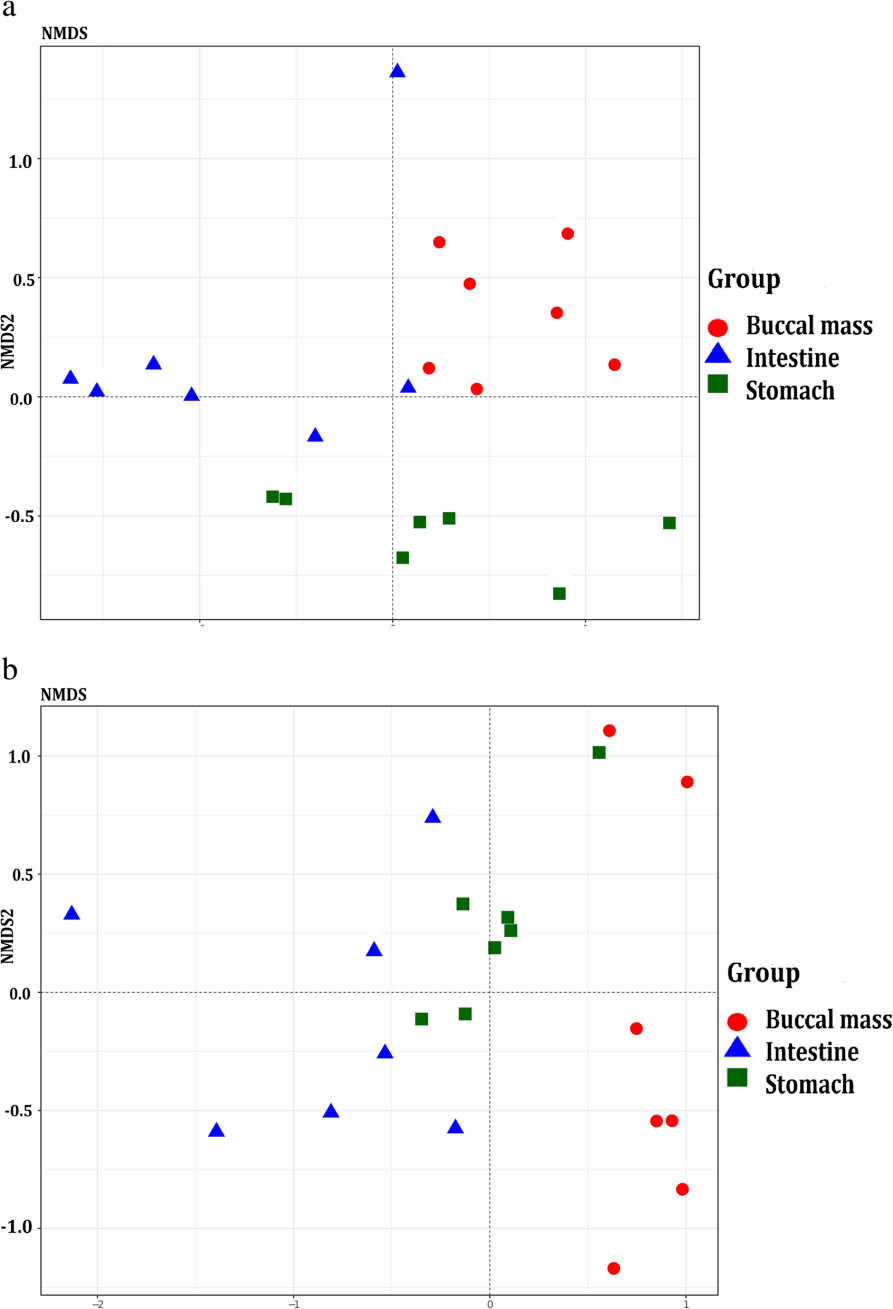
Two-dimensional distribution of samples according to (**a**) unweighted (**b**) weighted UniFrac distance-based NMS analysis

According to the results of the unweighted UniFrac distance-based NMS analysis, the intergroup distance was higher than the intragroup distance (Fig. 2a, Additional file 5: **Table S3).** Moreover, a similar pattern was confirmed by weighted UniFrac distance-based NMDS analysis (Fig. 2b, Additional file 6: **Table S4**). The results of ANOSIM also suggested that the intragroup similarity of the gut microbiome was different from the intergroup similarity (R = 0.5623, *P* = 0.001 for unweighted UniFrac distance; R = 0.4893, P = 0.001 for weighted UniFrac distance).

## Discussion

The gut microbiota of animals can play roles in the food ingestion, digestion and nutrient absorption of the host [8]. In snails, food is scraped by radula and mixed with the secretions of the salivary gland after being ingested by the buccal mass and digested in the stomach; nutrients are then absorbed in the intestine [9]. Little is known about the spatial structure of the gut microbiota in *P. canaliculata* snails. In this study, we assumed that the different gut sections, the buccal mass, stomach, and intestine, could be unique microenvironments and harbor distinct bacterial communities. To our knowledge, this is the first study to investigate the diversity and composition of bacterial communities in different gut sections of *P. canaliculata* snails. Our finding that the bacterial diversity was higher in the intestine (Table 1) is in agreement with reports from other animals [10]. This might be attributed to the characteristics that make the intestine more hospitable to bacteria than other regions of the gut [11].

Only a few studies have investigated the gut microbiota of snails. Moreover, most of these studies were not based on high-throughput sequencing. For example, Van and colleagues investigated intestinal bacterial communities in three species of planorbid snails (*Biomphalaria pfeifferi*, *Bulinus africanus*, and *Helisoma duryi*) via PCR amplification and sequencing of nearly full-length *16S* rRNA genes. They revealed that six bacterial taxa (*Aeromonas*, *Bacilli*, *Chryseobacterium*, *Chloroacidobacterium*, *Comamonadaceae* and *Verrucomicrobiae*) were present in at least one of the three snail species [12]. Kiran et al. studied cellulolytic bacteria from *Achatina fulica* using both culture and PCR product clones and sequencing of the 16S rRNA gene. They found that *Citrobacter*, *Escherichia*, *Klebsiella*, *Salmonella*, *Raoultella*, *Stenotrophomonas*, *Enterobacter*, and *Ochrobactrum* were present in the gastrointestinal tract [13].

Hu and colleagues investigated the gut microbiota of *Radix auricularia* via high-throughput sequencing of the *16S* rRNA gene. The results showed that unclassified genera of Mycoplasmataceae and Chloroflexaceae, *Paracoccus*, *Microcoleus*, *Pleurocapsa*, etc. were the most abundant genera [14]. In contrast, our study found that *Ochrobactrum*, *Sediminibacterium*, *Desulfovibrio*, an unclassified genus of Aeromonadaceae, and *Cloacibacterium* were dominant bacteria in the gut of *P. canaliculata* snails. The composition of the gut microbiota reflects natural selection between the bacteria and host, which promotes functional stability of this complex ecosystem [15]. The diverse composition of the gut microbiota in distinct snail species may be attributed to the differences in environments, habits, physiological states, genetic characteristics of the host, or the methods used for studying the bacterial community [16].

Bacteria with the potential to degrade plant components are common in the gut of snails. The present study showed that *Ochrobactrum* was most abundant in the stomach. *Ochrobactrum* bacteria are putative cellulose-degrading bacteria, which may play important roles in plant fiber digestion in herbivores [17]. They have also been reported in various ecological niches, including soil, water, plants, and animals [18], and have been reported in the human stomach [19] and the gut of *A. fulica* [13, 20]. Some researchers have suggested that the exogenous bacteria entering the intestinal tract of the snail with food have enzymatic activities that improve digestive processes [21]. In this study, *Ochrobactrum* was detected in all three gut sections of *P. canaliculata* snails, and it was the most abundant bacterium in the stomach. Moreover, another common bacterial composition from the gut of *P. canaliculata* snails, bacteria of the family Comamonadaceae, are also putative cellulose-degrading bacteria [12]. Koch et al. reported that *P. canaliculata* can survive 56 days on a cellulose-rich diet and suggested that bacterial endoglucanases could help the snail to utilize cellulose polymers [2]. However, whether the gut bacteria can degrade cellulose for the host cannot be inferred from the sequencing data [22]. More studies are still needed to further investigate the roles of putative cellulose-degrading bacteria in the gut of *P. canaliculata*.

Our study showed that the relative abundance of *Sediminibacterium* was higher in the stomach than in the buccal mass and intestine. *Sediminibacterium* is a common reagent contaminant [23]. However, the extraction products of the blank control failed to be amplified by PCR in this study. Therefore, it is reasonable to consider that the microbes reported in this study may not come from laboratory reagents. In previous studies, the genus *Sediminibacterium* was isolated from aquatic environments such as environmental water samples and sediments [24]. Similarly, *Sediminibacterium* was also detected from water samples (Fig. 1b). Therefore, *Sediminibacterium* in the gut was probably derived from the habitat of the snails.

Snails usually use copper for the formation of hemocyanin. *Desulfovibrio* spp. are sulfate-reducing bacteria and can chelate metals such as Cu, Fe, Ni, and Zn to enhance their absorption. *Desulfovibrio* has been reported in *Helix aspersa* crops [25]. In our study, *Desulfovibrio* was identified in all gut tissues. More studies are warranted to investigate whether *Desulfovibrio* plays a role in the metabolism of trace elements in *P. canaliculata* snails.

The relative abundance of *Leptotrichia* and bacteria from the family Porphyromonadaceae was significantly higher in the buccal mass than in the stomach and intestine of *P. canaliculata* snails. *Leptotrichia* is a facultative anaerobic bacterium and has been found mostly in the oral cavity and some other parts of the human body, in animals, and even in ocean sediments [26]. *Leptotrichia* species can ferment carbohydrates and produce lactic acid and might be associated with lactic acid metabolism in the buccal masses of snails [27]. Bacteria of the families Aeromonadaceae and Mollicute have been reported in various tissues of *A. fulica* and *H. pomatia*, respectively [16, 28]. Both of these bacterial taxa were more abundant in the intestine than in the buccal mass and stomach of *P. canaliculata* snails. *Cloacibacterium* was first described in 2006 and is usually found in wastewater [29]. It has also been isolated from sediment of freshwater lake [30] and the gut of abalone [31]. However, how these gut bacteria affect snail hosts remains to be investigated.

As a preliminary study on the gut microbiome of *P. canaliculata* snails, there are several limitations to the present study. First, bacterial DNA is ubiquitous in reagents and can cause problems when samples have a low microbial biomass [23, 32]. The extraction products of the blank control failed to be amplified by PCR in this study. Therefore, it is reasonable to consider that the microbes reported in this study may not come from laboratory reagents. However, contaminants from the environment cannot be excluded completely by surface sterilization of the shell.

Second, only three gut sections of seven *P. canaliculata* snails from one location were sequenced, and other snail species from the same ecological niche or the actual diet of the snails were not analyzed. Therefore, it is difficult to determine whether the bacteria detected from the snails are inherent or derived from the environment. In fact, most dominant bacteria detected in the gut of *P. canaliculata* snails existed in water samples at quite a low abundance (Fig. 1b) in this study. In another study, the composition and abundance of intestinal microbiota was found to be quite different in aquatic invertebrates collected from a single small pond [33]. These results suggested that certain microorganisms derived from the environment might be selectively colonized and established in the gut of the host. However, the gut microbiome of snails from different locations and other snail species needs to be analyzed in the future.

Third, *P. canaliculata* snails were dissected after starvation for 24 h in this study. Some researchers suggest that the bacterial community in the digestive tract of snails will be reduced to stable members after starvation [20]. However, it could have the opposite effect, for example, starvation may promote the growth of transient bacteria by inducing metabolic or immunological changes in the host. Therefore, the influence of starvation on the gut microbiome could not be determined. Since external factors, including diet, can largely affect the gut microbiota of the host [34], further investigation to compare the bacterial structure of snails that have and have not been starved is recommended. Moreover, the importance of the gut microbiota for the biology of the *P. canaliculata* snail cannot be inferred from the sequencing data and also requires further investigation.

## Conclusions

This study first describes the spatial structure of the microbiota in the gut of *P. canaliculata* snails using high-throughput sequencing. The results demonstrate that the diversity and composition of the microbiome vary among different gut sections of *P. canaliculata* snails. Putative cellulose-degrading bacteria, including *Ochrobactrum*, were abundant in the gut of *P. canaliculata*. More studies are required to better understand the interaction between the gut microbiota and its snail host, including *P. canaliculata*.

## Methods

### Sample collection and tissue processing

*P. canaliculata* snails were collected from Nanheng River (31.052649°N, 120.99297°E) in Rentun village, Qingpu district, Shanghai, PR China, in July 2018. Prior to dissection, the snails were starved for 24 h to minimize the amount of partially digested food in the gut [12]. Simultaneously, three water samples were collected from the habitat of the snails using sterilized bottles and transferred to the laboratory immediately. Water samples were concentrated using 0.22 μm polyether sulfone membrane filters (Millipore, Darmstadt, Germany). The filtration volume was one liter per sample [35]. The membranes coated with microbes from the water samples were used for DNA extraction. The membranes were cut into small pieces and homogenized in SLX-Mlus Buffer (Omega, Norcross, United States) using a Tissuelyser (Jingxin Industrial development Co., Ltd., Shanghai, China). DNA was extracted from the homogenate using the Mag-Bind Soil DNA Kit M5635–02 (Omega, Norcross, U.S.A.) following the manufacturer’s protocol.

**Fig. 3:**
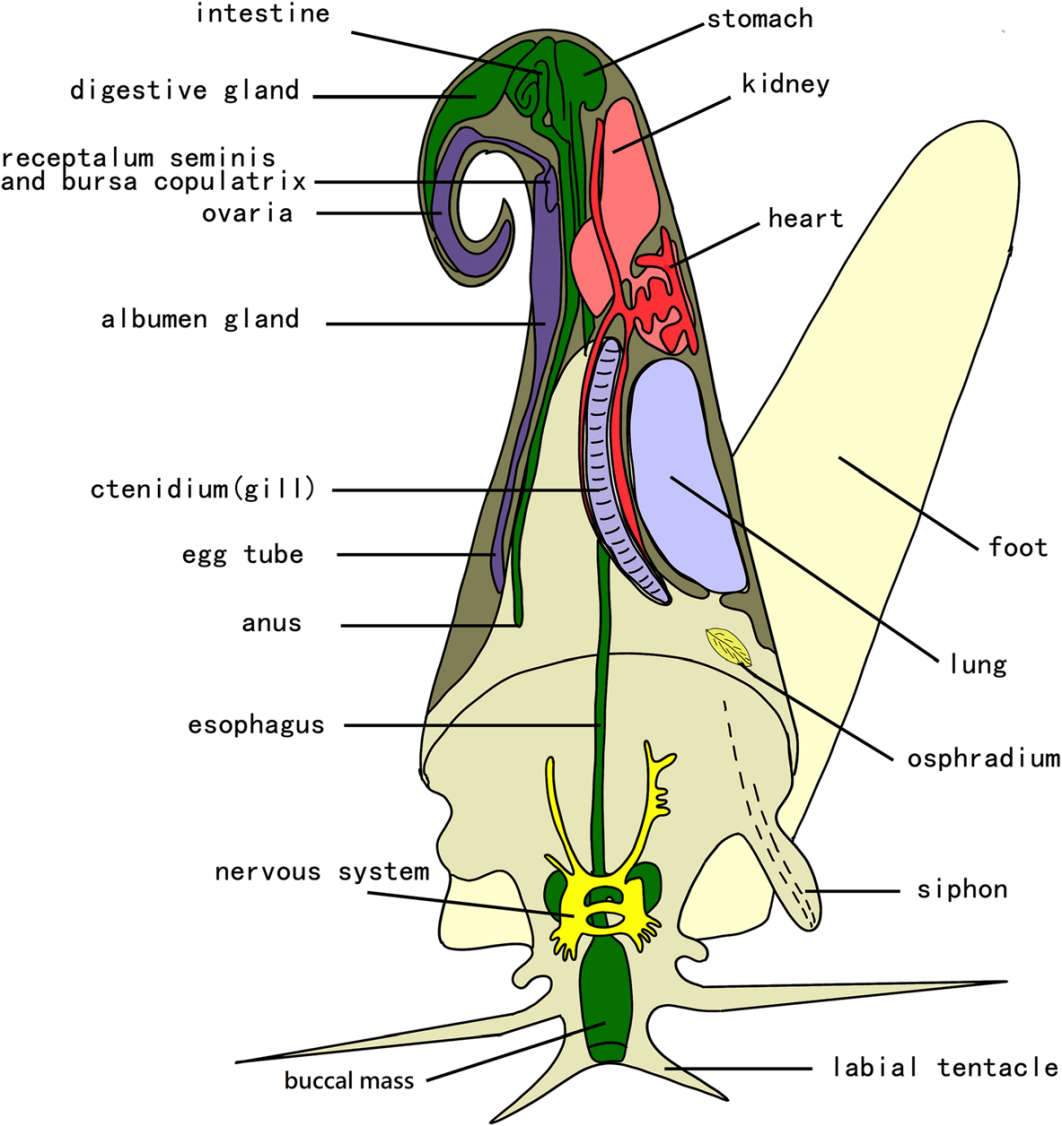
The simplified anatomic diagram of *Pomacea canaliculata* (www.applesnail.net, by Dr. Stijn Ghesquiere. We thank Dr. Stijn Ghesquiere for permission to use the diagram)

Seven female *P. canaliculata* snails weighing 9–10 g were selected for dissection. The shell was removed from each snail after wiping the shell with 70% ethanol three times and rinsing it twice in distilled water. Dissection was performed on ice in sterile Petri dishes using flame-sterilized tools. The buccal mass, stomach, and intestine of each snail were isolated (Fig. 3) and homogenized separately in centrifuge tubes with a Tissuelyser. DNA was extracted from the homogenized tissue using the Mag-Bind Soil DNA Kit M5635–02 as described above. To exclude contaminants from reagents, three blank controls were extracted simultaneously using the same DNA extraction kit.

### Sequencing of the microbial *16S* rRNA genes

The variable V3-V4 region of the *16S* rRNA gene was amplified by PCR using universal bacterial primers (338F: 5′-ACTCCTACGGGAGGCAGCA-3′, 806R: 5′-GGACTACHVGGGTWTCTAAT-3′). PCR amplification was performed with an ABI 2720 thermal cycler (Applied Biosystems, Foster City, United States) in a total volume of 25 μL containing 8.75 μL of ddH_2_O, 5 μL of 5× reaction buffer, 5 μL of 5× GC buffer, 2 μL of dNTPs (2.5 mM), 1 μL of each primer (10 μM), 2 μL of DNA template, and 0.25 μL of Q5 High-Fidelity DNA Polymerase (NEB, Ipswich, UK). The thermal cycling conditions were an initial denaturation at 98 °C for 5 min, followed by 25 cycles of 98 °C for 30 s, 55 °C for 45 s and 72 °C for 30 s, and a final extension at 72 °C for 5 min. The PCR products were detected by 2% agarose gel electrophoresis and purified with an AxyPrep DNA Gel Extraction Kit (Axygen, New York, United States). The purified PCR amplicons were used to construct paired-end DNA libraries using the TruSeq Nano DNA LT Library Prep Kit (Illumina, San Diego, United States). Each PCR product was tagged with an index sequence at the 5′ end of the forward primer and then sequenced on the Illumina MiSeq platform (300 bp paired-end reads) by Personal Biotechnology Co., Ltd. (Shanghai, China).

### Sequencing data analysis

Quantitative Insights Into Microbial Ecology (QIIME) software (v1.8.0) was used to process the raw sequences. Reads containing any ambiguous bases, sequences shorter than 150 bp, or chimeric sequences were removed. All of the trimmed sequences were normalized to the same sequencing depth using the Mothur software package (v.1.31.2) [36]. The operational taxonomic units (OTUs) were clustered at 97% identity using the UCLUST tool of QIIME software [37]. The sequence with the highest abundance was selected as a representative sequence of each OTU. The taxonomy of each representative sequence was assigned according to the Greengenes 13.8 database [38]. The original OTU abundance matrix usually contains a large number of OTUs with very low abundance, which often occurs occasionally in a small number of samples (i.e., low frequency), while the number of OTUs with high abundance is relatively small. Those rare OTUs with very low abundance and frequency can greatly increase the complexity of data analysis. Removing these rare OTUs has little effect on the diversity of the bacterial community but can significantly improve the efficiency of data analysis. Therefore, OTUs with relative abundance less than 0.001% of all OTUs were removed prior to analysis [39].

A rarefaction curve was drawn to determine whether the sequencing depth was sufficient to represent the bacterial diversity of each sample using the Mothur software package [40]. Alpha diversity indices of the gut microbiome, including the ACE, Chao1, Shannon and Simpson indices, were estimated using QIIME.

To estimate the beta diversity or similarity of the gut microbiome among tissues, nonmetric multidimensional scaling (NMDS) analysis was performed to visualize the pairwise UniFrac distances among samples using R software based on unweighted and abundance weighted UniFrac distance [41]. The tests of significance between intragroup and intergroup UniFrac distance were performed using the Monte Carlo permutation test with QIIME software. Analysis of similarity (ANOSIM) was further conducted to analyze the differences between the intragroup and intergroup distances.

SPSS 19.0 software (IBM, Armonk, USA) was used for statistical analysis of the alpha diversity, number of OTUs, and relative abundance of bacterial taxa among groups using one-way ANOVA. A *P*-value less than 0.05 was considered statistically significant.

## Supplementary information

**Additional file 1: Fig. S1.** The rarefaction curve of observed species in the samples.

**Additional file 2: Fig. S2.** Venn diagram of shared OTUs among different gut sections of *P. canaliculata.*

**Additional file 3: Table S1.** The relative abundance of the top 10 phyla of the gut microbiome in the three gut sections of *P. canaliculata*.

**Additional file 4: Table S2.** The relative abundance of the top 10 genera of the gut microbiome in the three gut sections of *P. canaliculata.*

**Additional file 5: Table S3.** Differences between intragroup and intergroup unweighted UniFrac distances.

**Additional file 6: Table S4.** Differences between intragroup and intergroup weighted UniFrac distances.

## Data Availability

The sequences generated in the present study were deposited in the NCBI Sequence Read Archive (nos. SRR9166284 - SRR9166304).

## Abbreviations

ANOSIM: Analysis of similarity
ANOVA: Analysis of variance
LEfSe: Linear discriminant analysis effect size
NMS: Nonmetric multidimensional scaling
OTU: Operational taxonomic unit
