# Supplementary figures and images for: Spatial structure of the microbiome in the gut of Pomacea canaliculata
Source: BMC Microbiol. 2019 Dec 5;19:273. doi: 10.1186/s12866-019-1661-x (PMC6896589; doi:10.1186/s12866-019-1661-x)

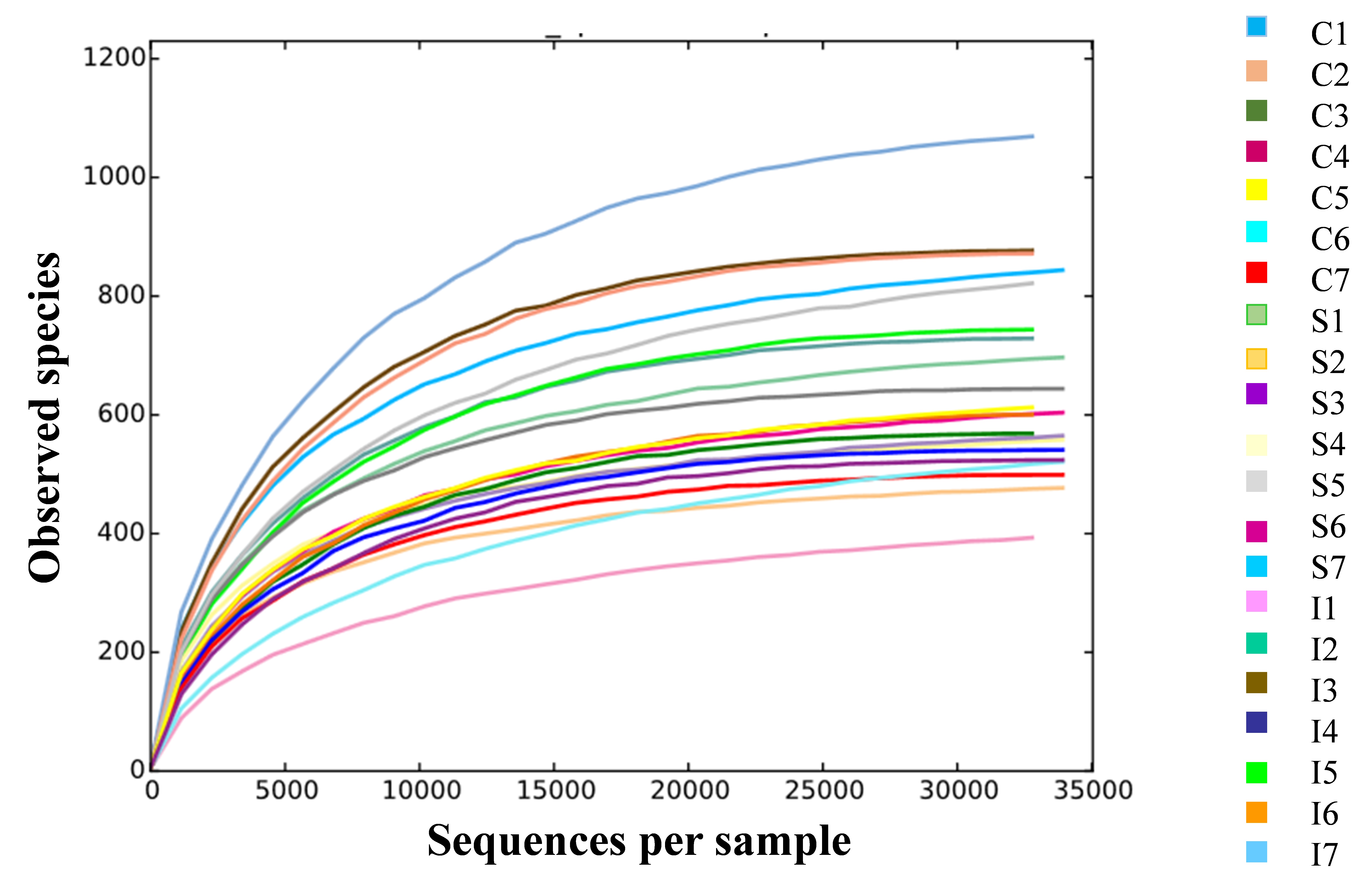

Supplement: Supplementary file 1 — Additional file 1: Fig. S1. The rarefaction curve of observed species in the samples. [file 12866_2019_1661_MOESM1_ESM.jpg]

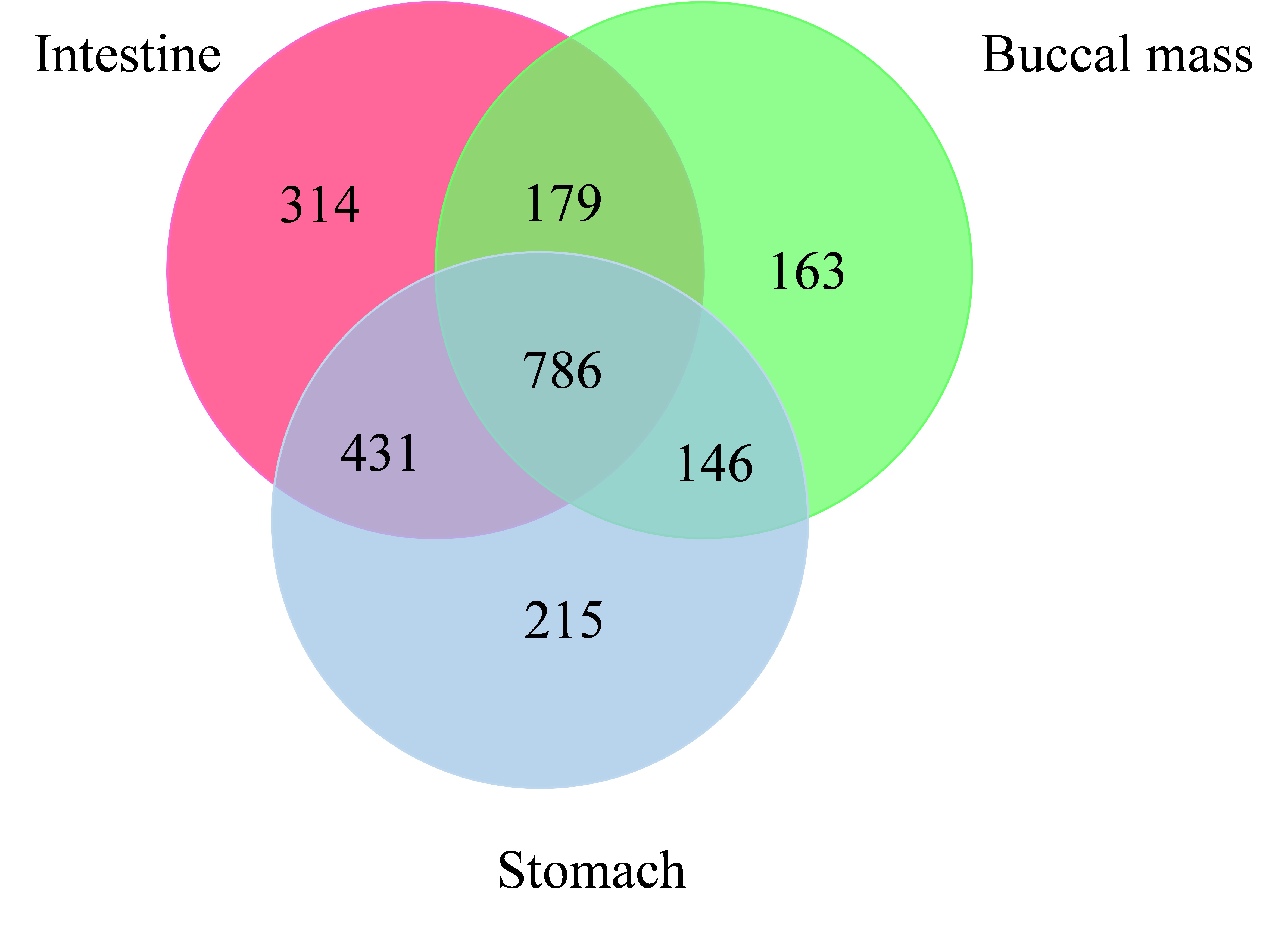

Supplement: Supplementary file 2 — Additional file 2: Fig. S2. Venn diagram of shared OTUs among different gut sections of P. canaliculata. [file 12866_2019_1661_MOESM2_ESM.jpg]
